# Imaging the scattered light of a nanoparticle through a cylindrical capillary

**DOI:** 10.1515/nanoph-2023-0773

**Published:** 2024-01-29

**Authors:** Ulrich Hohenester, Christian Neuper, Marko Šimić, Christian Hill

**Affiliations:** Institute of Physics, University of Graz, Graz, Austria; Brave Analytics GmbH, Graz, Austria; Graz Centre for Electron Microscopy, Graz, Austria; Gottfried Schatz Research Center, Division of Biophysics, Medical University of Graz, Graz, Austria

**Keywords:** optical microscopy, Mie scattering, Richards–Wolf approach, optofluidic force induction

## Abstract

In many experiments, nanoparticles are located inside a microfluidic channel, and the light scattered by the particles becomes diffracted through the walls of the capillary. We here derive a simple but accurate approach for simulating the imaging of light through a cylindrical capillary under the assumption that the dimensions of the capillary are much larger than the wavelength of light. A comparison of the simulated images with experimental results shows very good agreement.

## 1 Introduction

Optical detection of nanoparticles in microfluidic channels plays an important role for various nanoparticle characterization schemes [1], [2], [3], [4], [5], [6]. Through the walls of the capillary that encapsulates the flow channel, the detected light becomes diffracted, and without further correction, the images of the particles are lines rather than points [4]. A pair of cylindrical lenses in the detection path can be used for a partial compensation of this astigmatism [1].

For a better understanding and a possible optimization of the whole imaging process, a simulation of the consecutive light scattering, diffraction, and imaging is needed. However, the different length scales of the nanoparticle and the capillary, whose diameter is typically in the millimeter range, make a full vectorial wavefunction analysis difficult and computationally expensive.

In this paper, we present a simple but accurate description for the imaging of light scattered by nanoparticles inside a cylindrical capillary, which fully exploits the cylinder symmetry of the problem under study. The main assumption is that the dimensions of the capillary are much larger than the wavelength of light, such that only the scattered far-fields have to be considered. With this, we derive analytic expressions for the scattered light, which, to the best of our knowledge, have not been derived before. We also compare our simulation results with supplementary experiments, finding very good agreement throughout.

**Figure 1:**
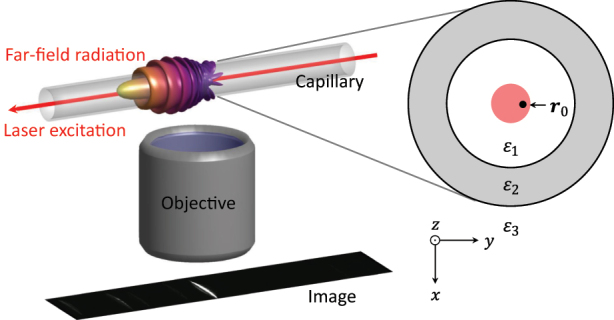
Schematics of imaging. A nanoparticle is located at position ***r***_0_ close to the center of the capillary and is optically excited by an incoming laser. The scattered light is imaged through a microscope objective.

The setup we have in mind is depicted in Figure 1. A nanoparticle is situated at position ***r***_0_, close to the symmetry axis of the capillary. In our theoretical approach, we trace the radiation from the nanoparticle through the walls of the capillary to the imaging system, which we describe with the approach of Richards and Wolf [7]. This is done in four steps. (i) The starting point of our calculation is formed by the multipole coefficients for the scattered light [[8], Section 9.7], which can be obtained for instance from Mie theory or a computational Maxwell solver. (ii) We next translate the coefficients from spherical to cylinder coordinates and shift the coefficients from the center position of the nanoparticle to the origin of the cylinder coordinate system. Finally, (iii) we trace the scattered fields through the cylindrical capillary, using a transfer matrix approach together with the usual Fresnel coefficients, before (iv) submitting them to the Richards–Wolf imaging procedure. In the following, we discuss the various steps in slightly more detail.

## 2 Theory

For the electromagnetic fields scattered by the nanoparticle, we use the basis of transverse vector wave functions [[9], Section 7.2]

(1) $$\begin{array}{l} \begin{array}{cc} M\left(r\right) & =\nabla \times c\;\psi \left(r\right)\\ N\left(r\right) & =\frac{1}{k}\nabla \times M\left(r\right). \end{array} \end{array}$$

Here, ***c*** is a pilot vector, to be specified in a moment, *ψ*(***r***) is a function that fulfills the scalar wave equation, and *k* is the wavenumber of light in the embedding medium of the nanoparticle. With the boundary condition of outgoing waves at infinity and for spherical coordinates, the pilot function is ***r*** and *ψ* is composed of spherical Hankel functions and spherical harmonics [[9], Eq. (7.2.41)]

(2) $$c\;\psi \left(r\right)=r\;h_{\ell }^{\left(1\right)}\left(kr\right)Y_{\ell m}\left(\theta ,\phi \right).$$

Here, *ℓ* and *m* denote the spherical degrees and orders, respectively, and $h_{\ell }^{\left(1\right)}$ is the spherical Hankel function of first order [[8], Eq. (9.85)]. Similarly, for cylinder coordinates, the pilot function is $\hat{z}$ and *ψ* is composed of Hankel functions [[9], Eq. (7.2.32)]

(3) $$c\;\psi \left(r\right)=\frac{i}{2k}\hat{z}H_{m}^{\left(1\right)}\left(k_{\rho }\rho \right)e^{im\phi }e^{ik_{z}z},$$

with *k*_*ρ*_ = *k* sin *α* and *k*_*z*_ = *k* cos *α*, where *α* is an angle. The prefactor has been chosen for later convenience. For spherical coordinates, the scattered electromagnetic fields can now be expressed in the form

(4) $$\begin{array}{l} \begin{array}{cc} E & =Z\underset{\ell ,m}{\sum }\left[b_{\ell m}M_{\ell m}\left(r\right)+ia_{\ell m}N_{\ell m}\left(r\right)\right]\\ H & =\underset{\ell ,m}{\sum }\left[a_{\ell m}M_{\ell m}\left(r\right)-ib_{\ell m}N_{\ell m}\left(r\right)\right], \end{array} \end{array}$$

where *Z* is the impedance of the embedding medium. Similarly, for cylinder coordinates, we get

(5) $$\begin{array}{l} \begin{array}{cc} & E=Z\underset{m}{\sum }\overset{\pi }{\underset{0}{\int }}\left[b_{m}\left(\alpha \right)M_{m}\left(\alpha ,r\right)+ia_{m}\left(\alpha \right)N_{m}\left(\alpha ,r\right)\right]\;d\alpha \\ & H=\underset{m}{\sum }\overset{\pi }{\underset{0}{\int }}\left[a_{m}\left(\alpha \right)M_{m}\left(\alpha ,r\right)-ib_{m}\left(\alpha \right)N_{m}\left(\alpha ,r\right)\right]\;d\alpha . \end{array} \end{array}$$

Suppose that we are in possession of the spherical multipole coefficients *a*_*ℓm*_, *b*_*ℓm*_, e.g., from the solution of Mie theory for spherical nanoparticles [5]. We are now seeking for the coefficients *a*_*m*_(*α*), *b*_*m*_(*α*), which we obtain from *a*_*ℓm*_, *b*_*ℓm*_ by matching the optical far-fields. The far-field approximation of Eq. (4) is Ref. [[8], Eq. (9.149)]

(6) $$H\to \frac{e^{\mathrm{ikr}}}{kr}\underset{\ell ,m}{\sum }{\left(-i\right)}^{\ell +1}\left[a_{\ell m}X_{\ell m}+b_{\ell m}\hat{r}\times X_{\ell m}\right],$$

where ***X***_*ℓm*_ are the vector spherical harmonics. In the far-field zone, the electric and magnetic fields are related through $E=ZH\times \hat{r}$ , with the propagation direction $\hat{r}$ of the wave. Similarly, as outlined in more detail in Section 5.1, the far-field approximation of Eq. (5) is

(7) $$H\to \frac{e^{\mathrm{ikr}}}{kr}\underset{m}{\sum }{\left(-i\right)}^{m+1}\left[a_{m}\left(\theta \right)\hat{\phi }+b_{m}\left(\theta \right)\hat{r}\times \hat{\phi }\right]e^{im\phi },$$

where *ϕ*, *θ* are the azimuthal and polar angles of the propagation direction $\hat{r}$ , respectively, and $\hat{\phi }$ is the unit vector in the azimuthal direction. We next note that in both Eqs. (6) and (7), the fields have a *e* ^*imϕ*^ dependence for the azimuthal angle. By taking the inner product of Eqs. (6) and (7) and the corresponding expressions for the electric field with $\hat{\phi }$ , we can relate the spherical and cylinder expansion coefficients through

(8) $$\begin{array}{l} \begin{array}{cc} a_{m}\left(\theta \right) & =\underset{\ell }{\sum }{\left(-i\right)}^{\ell +m}\left[a_{\ell m}\;\hat{\phi }\cdot X_{\ell m}+b_{\ell m}\;\hat{\phi }\cdot \hat{r}\times X_{\ell m}\right]\\ b_{m}\left(\theta \right) & =\underset{\ell }{\sum }{\left(-i\right)}^{\ell +m}\left[b_{\ell m}\;\hat{\phi }\cdot X_{\ell m}-a_{\ell m}\;\hat{\phi }\cdot \hat{r}\times X_{\ell m}\right], \end{array} \end{array}$$

where in all expressions, the vector spherical harmonics have to be evaluated for *ϕ* = 0. Eq. (8) allows us to convert the expansion coefficients from spherical to cylinder coordinates.

The next step is optional and is needed when the nanoparticle is not located at the origin of the coordinate system. If the expansion coefficients *a*_*ℓm*_, *b*_*ℓm*_ are already computed for the shifted particle, this step can be omitted. However, if the coefficients are computed for the particle center as the origin, the expansion coefficients need to be translated. This can be done either for the spherical or cylinder coefficients, where we discuss the latter case only. Suppose that we shift the positions from ***r*** → ***r*** + ***r***_0_. The exponential factor in Eq. (7) then changes accordingly [[8], Eq. (9.7)]

(9) $$\frac{e^{\mathrm{ikr}}}{r}\to \left(\frac{e^{\mathrm{ikr}}}{r}\right)e^{-ik\left[\rho_{0}\sin\theta \cos\left(\phi -\phi_{0}\right)+z_{0}\cos\theta \right]},$$

where in the second term on the right hand side, we have expressed $e^{-ik\hat{r}\cdot r_{0}}$ in cylinder coordinates. The shifted expansion coefficients are obtained by re-expanding the shifted far-fields into a Fourier series viz.

(10) $$\begin{array}{ll} a_{m} & \to e^{-ik_{z}z_{0}}\underset{m^{\prime }}{\sum }{\left(-i\right)}^{m^{\prime }-m}\\ & \;\times \overset{2\pi }{\underset{0}{\int }}{\text{e}}^{-\text{i}\left(m-m^{\prime }\right)\phi }e^{-ik_{\rho }\rho_{0}\cos\left(\phi -\phi_{0}\right)}a_{m^{\prime }}\;\frac{d\phi }{2\pi }, \end{array}$$

where we have introduced *k*_*ρ*_ = *k* sin *θ* and *k*_*z*_ = *k* cos *θ*. The integral can be solved analytically [[10], Eq. (3.21)], and we obtain

(11) $$a_{m}\to e^{-ik_{z}z_{0}}\underset{m^{\prime }}{\sum }J_{m-m^{\prime }}\left(k_{\rho }\rho_{0}\right){\text{e}}^{-\text{i}\left(m-m^{\prime }\right)\phi_{0}}a_{m^{\prime }},$$

with the Bessel function *J*_*m*−*m*′_. Eq. (11) is the central equation for translating the expansion coefficients when shifting the origin of the coordinate system. A corresponding expression holds for *b*_*m*_. Note that the summation in Eq. (11) has the form of a convolution and can be computed efficiently by means of a fast Fourier transform.

We next propagate the far-fields of Eq. (7) through the cylindrical capillary. The light wavelength is assumed to be much shorter than the diameter of the capillary, which is typically of the order of millimeters, and we correspondingly use the far-field limit for the electromagnetic fields. Additionally, we consider multiple reflections of the scattered fields only within the glass wall of the capillary but ignore the reflected fields at the interface between the inner core and glass. These fields could be treated along the same line as discussed in Section 5.2; however, considering the minor importance of multiple reflections already inside the glass boundary, we think that our neglect is well justified. The coefficients $a_{m}^{\left(1\right)}$ in the core region can then be related to the coefficients outside the capillary according to

(12) $$a_{m}^{\left(3\right)}\left(\theta \right)=\frac{n_{3}}{n_{1}}\sqrt{\frac{k_{1\rho }}{k_{3\rho }}}{\text{e}}^{\text{i}\psi }\frac{T_{12}T_{23}}{1-R_{21}R_{23}e^{2i\psi_{2}}}a_{m}^{\left(1\right)}\left(\theta \right).$$

Here, *R* and *T* are the transverse magnetic reflection and transmission coefficients between the different regions, respectively, and *ψ *and* 2iψ* are propagation constants listed in Section 5.2. There we give further details about the derivation of Eq. (12) and discuss how to obtain a corresponding expression for the coefficients *b*_*m*_(*θ*).

In the last step, we image the scattered fields using the approach of Richards and Wolf [7], which is based on the assumption that the imaging objective has perfect aplanatic properties. As input for this approach, we need the scattered far-field amplitudes ***F*** (*θ*, *ϕ*) at the surface of the Gaussian reference sphere, where the opening angle *θ*_max_ is given by the numerical aperture of the objective. Using a coordinate system where the *z* axis coincides with the optical axis, the imaged electric field is given by [[10], Eq. (3.10)]

(13) $$\begin{array}{ll} E\left(\rho ,\varphi ,z\right) & \propto \overset{\theta_{\max}}{\underset{0}{\int }}\sqrt{\cos\theta }\sin\theta d\theta \overset{2\pi }{\underset{0}{\int }}d\phi \\ & \;\times \bar{\bar{R}}\cdot F\left(\theta ,\phi \right)e^{-ik_{3}\cos\theta z}e^{ik_{3}\frac{\rho }{M}\sin\theta \cos\left(\phi -\varphi \right)}. \end{array}$$

Here, $\bar{\bar{R}}$ is a matrix accounting for the change of the coordinate system for waves propagating through the lens, *k*_3_ is the wavenumber in front of the lens, and *M* is the lens magnification. We use coordinate systems *θ*, *ϕ* and *ρ*, *φ*, *z* in front and behind the lens. The evaluation of Eq. (13) can be accelerated significantly by using a fast Fourier transformation, as described in more detail in Ref. [10].

## 3 Results

**Figure 2:**
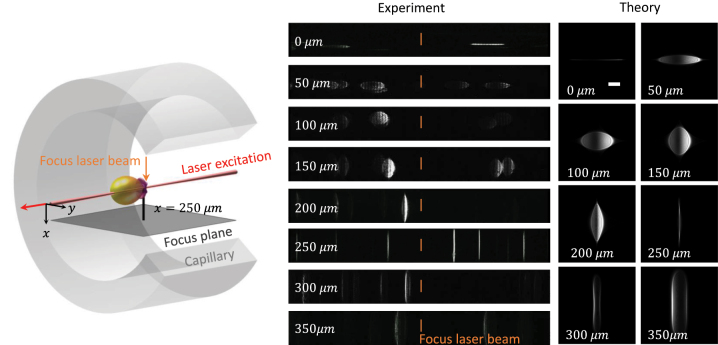
Experimental and theoretical images for nanoparticle scattering through a cylinder capillary. In the of2i experiments, polystrene nanoparticles (refractive index 1.59, diameter 600 nm) are transported through a flow cell (flow from right to left) and are excited from the right-hand side by a weakly focused Laguerre–Gauss laser beam with a polarization along *y*. The experimental images show snapshots of the nanoparticles in the focus region *z* ≈ 0 of the Gauss–Laguerre beam and for different focus plane positions *x* of the side camera reported in the insets, see also Figure 1 and discussion in text. The theoretical images are obtained using the formalism developed in this paper and for trapped particles at *z* = 0. We use parameters representative for experiment [4], [5]. The scale bar in the theory panel is 50 μm.

To test the formalism developed in this paper, in Figure 2, we compare the results of our simulations with optofluidic force induction (of2i) experiments [4], [5], [11]. of2i is a nanoparticle characterization scheme where nanoparticles are transported through a microfluidic channel alongside a weakly focused Laguerre–Gauss beam (wavelength 532 nm, topological charge *m* = 2), which traps the nanoparticles in the transverse directions *x*, *y* and exerts forces on the particles. This can be used to determine the sizes and size distributions of the particles, see Refs. [4], [5], [11] for details. For our present purpose, the only important point is that the particles are excited by the laser, which propagates along the cylinder axis, and the light scattered by the particles and diffracted by the walls of the cylinder is imaged in the perpendicular direction, see Figure 1.

In our theoretical approach, we take the same laser parameters as in Refs. [4], [5], [11] and consider in accordance to the experiment polystrene nanoparticles (refractive index 1.59) with a diameter of 600 nm. For the refractive indices indicated in Figure 1, we use *n*_1_ = 1.33, *n*_2_ = 1.5, and *n*_3_ = 1 representative for water, glass, and air, respectively, and a cylindrical glass capillary with inner and outer diameters of 1.3 and 1.8 mm, respectively. In the of2i experiments, the working point is for the focus plane *x* = 250 μm where the nanoparticles are imaged as vertical lines. When shifting the focus plane toward the symmetry axis of the cylinder, the lines broaden and change shape, until one ends up with a horizontal line. Comparison with theory shows that here the symmetry axis lies exactly in the focus plane. We observe that the experimental and theoretical images are in very good agreement throughout, thus showing that our approach indeed captures the main features of corresponding experiments. We also found that multiple reflections in the glass capillary are of minor importance, when replacing the denominator in Eq. (12) with one we obtained practically the same results.

Note that in Figure 2, we show for the of2i experiments snapshots where multiple polystrene nanoparticles are located at different propagation distances *z* along the flow channel. Close to the focus *z* = 0 of the Gauss–Laguerre beam the images do not depend decisively on the propagation distance *z*, as can be seen by comparing the images of the different particles. The minor differences are due to the different trapping positions of the nanoparticles in the transverse directions, owing to the ring-shaped intensity profile of the Gauss–Laguerre beam [5], [12]. For the laser excitation, we consider in accordance to Refs. [4], [5] a linear polarization along *y*, see inset of Figure 2. However, our approach can be safely applied to other types of laser excitations without any modifications once the multipole coefficients *a*_*ℓm*_, *b*_*ℓ*,*m*_ are at hand.

**Figure 3:**
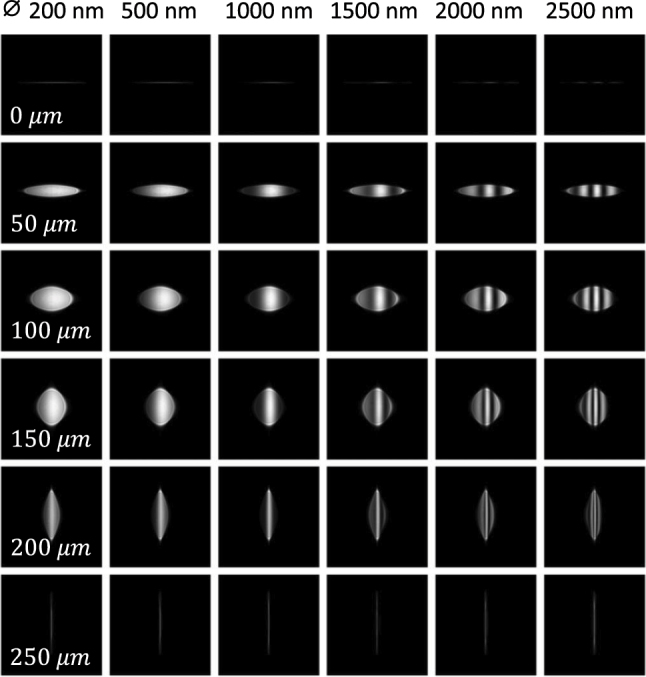
Images of nanoparticles for different focus planes (see insets) and diameters (reported on top). We use the same simulation, material, and laser parameters as in Figure 2.

Finally, in Figure 3, we show nanoparticle images for different focus planes and nanoparticle diameters, using the same laser and nanoparticle parameters as in Figure 2. The images exhibit stripe-like structures that can be traced back to the far-field emission patterns of Mie scattering, see Figure 1, which could serve in future experiments as fingerprints for the particle diameters.

## 4 Summary

To summarize, we have presented a simple approach for the imaging of light scattered by a nanoparticle inside a cylindrical capillary. It combines a matching of far-fields in spherical and cylindrical coordinates, a transfer matrix approach for the light propagation through the walls of the cylinder, together with the Richards–Wolf approach for imaging. Similar results could be obtained from more detailed simulation approaches, such as ray tracing, but the appealing features of our scheme are its simplicity, the elegant combination of analytic methods, and the resulting computational speed. We have also shown that the results of our approach agree well with experiment. We think that our results could be useful for other nanoparticle characterization or localization schemes, such as nanoparticle tracking analyis (nta) or interference scattering (iscat), or for benchmarking more complete simulation approaches where the consideration of interference between all scattered field components constitutes a formidable computational challenge.

## 5 Methods

### 5.1 Far-field limit of wave function

We here show how to compute the far-field limit of Eq. (5). The limit of the Hankel function for large arguments is

(14) $$H_{m}^{\left(1\right)}\left(x\right)\to \sqrt{\frac{2}{\pi x}}{\left(-i\right)}^{m}e^{ix-i\frac{\pi }{4}}.$$

Correspondingly, we obtain for the scalar function in Eq. (3)

$$\psi \left(r\right)\to \frac{1}{\sqrt{2\pi k^{3}\rho \sin\alpha }}{\left(-i\right)}^{m-\frac{1}{2}}e^{im\phi }e^{ik\left(\rho \sin\alpha +z\cos\alpha \right)}.$$

The transverse vector function is then obtained from

$$\nabla \times \hat{z}\psi =\frac{\partial \psi }{\partial \rho }\hat{\rho }\times \hat{z}+\frac{1}{\rho }\frac{\partial \psi }{\partial \phi }\hat{\phi }\times \hat{z}\to -\frac{\partial \psi }{\partial \rho }\hat{\phi },$$

where in the last limit, we have only kept the contribution that decays slowest with respect to *ρ*. With this, we readily obtain the far-field limit of the transverse vector function

(15) $$M\to \sqrt{\frac{\sin\alpha }{2k\pi \rho }}{\left(-i\right)}^{m+\frac{1}{2}}e^{im\phi }e^{ik\left(\rho \sin\alpha +z\cos\alpha \right)}\;\hat{\phi }.$$

Using the shorthand notation $M\to M\hat{\phi }$ for the above expression, a similar analysis gives

$$\nabla \times M\hat{\phi }=-\frac{\partial M}{\partial z}\hat{\rho }+\frac{\partial M}{\partial \rho }\hat{z}\to -ikM\left(\cos\alpha \;\hat{\rho }-\sin\alpha \;\hat{z}\right),$$

which allows computing the far-field limit of the second transverse vector function. We next express in spherical coordinates *ρ* = *r* sin *θ* and *z* = *r* cos *θ* and use sin *α* sin *θ* + cos *α* cos *θ* = cos(*α* − *θ*). The integrals in Eq. (5) are then of the form

(16) $$I=\overset{\pi }{\underset{0}{\int }}f\left(\alpha \right)e^{ikr\cos\left(\alpha -\theta \right)}\;d\alpha ,$$

where *f*(*α*) is a function of the angle *α* and of the position ***r***. We next explore the far-field limit of Eq. (16). For large values of *kr*, the integrand in Eq. (16) oscillates wildly and one can employ the stationary phase approximation [[10], Section 3.2]

$$\overset{2\pi }{\underset{0}{\int }}f\left(\alpha \right)e^{ikrg\left(\alpha \right)}\;d\alpha \to \sqrt{\frac{2\pi }{kr|g^{\prime \prime }\left(\alpha_{0}\right)|}}f\left(\alpha_{0}\right)e^{ikrg\left(\alpha_{0}\right)\pm i\frac{\pi }{4}},$$

where the dominant contribution to the integral is for the angle *α*_0_ where *g*(*α*) has a maximum (negative sign) or minimum (positive sign). With this, the integral of Eq. (16) evaluates to

(17) $$I\to \sqrt{\frac{2\pi }{kr}}\;f\left(\theta \right)e^{ikr-i\frac{\pi }{4}}.$$

Putting together all results and using Eq. (17), we arrive at our final expression of Eq. (7). Note that the prefactor in Eq. (3) has been chosen such that the far-fields for spherical and cylinder coordinates in Eqs. (6) and (7) have similar prefactors.

### 5.2 Field propagation through capillary

We here show how to propagate the fields from the core region through the capillary. We first consider the term proportional to *a*_*m*_(*θ*) in the brackets of Eq. (7), which is parallel to the cylinder interfaces and has a transverse magnetic character. It can be propagated through the capillary using the usual Fresnel coefficients. Together with Eq. (15), the magnetic field component for a given angular order *m* is apart from a constant prefactor

(18) $$H_{1}^{\left(+\right)}\left(\rho \right)={\left(\frac{k_{1\rho }}{k_{1}^{2}\rho }\right)}^{\frac{1}{2}}{\text{e}}^{\text{i}\left(m\phi +k_{z}z\right)}e^{ik_{1\rho }\rho }a_{m}^{\left(1\right)}\hat{\phi }.$$

Similarly, the field components in the glass region consist of an outgoing and ingoing cylindrical wave, and the component in the outer region of an outgoing wave

$$\begin{array}{ll} H_{2}^{\left(+\right)}\left(\rho \right) & ={\left(\frac{k_{2\rho }}{k_{2}^{2}\rho }\right)}^{\frac{1}{2}}{\text{e}}^{\text{i}\left(m\phi +k_{z}z\right)}e^{+ik_{2\rho }\rho }a_{m}^{\left(2\right)}\hat{\phi }\\ H_{2}^{\left(-\right)}\left(\rho \right) & ={\left(\frac{k_{2\rho }}{k_{2}^{2}\rho }\right)}^{\frac{1}{2}}{\text{e}}^{\text{i}\left(m\phi +k_{z}z\right)}e^{-ik_{2\rho }\rho }d_{m}^{\left(2\right)}\hat{\phi }\\ H_{3}^{\left(+\right)}\left(\rho \right) & ={\left(\frac{k_{3\rho }}{k_{3}^{2}\rho }\right)}^{\frac{1}{2}}{\text{e}}^{\text{i}\left(m\phi +k_{z}z\right)}e^{+ik_{3\rho }\rho }a_{m}^{\left(3\right)}\hat{\phi }. \end{array}$$

Note that in the above expressions, *k*_*z*_ is a conserved quantity, and the radial wavevector components in the different media can be computed from $k_{n}^{2}=k_{n\rho }^{2}+k_{z}^{2}$ . In order to obtain the unknown coefficients, we relate the fields at the interfaces through

$$\begin{array}{ll} H_{2}^{\left(+\right)}\left(R_{1}\right) & =T_{12}H_{1}^{\left(+\right)}\left(R_{1}\right)+R_{21}H_{2}^{\left(-\right)}\left(R_{1}\right)\\ H_{2}^{\left(-\right)}\left(R_{2}\right) & =R_{23}H_{2}^{\left(+\right)}\left(R_{2}\right)\\ H_{3}^{\left(+\right)}\left(R_{2}\right) & =T_{23}H_{2}^{\left(+\right)}\left(R_{2}\right). \end{array}$$

Here, *R* and *T* are the transverse magnetic reflection and transmission coefficients. The first equation states that $H_{2}^{\left(+\right)}$ is either created through a transmission of the outgoing wave in medium 1 or a reflection of the ingoing wave in medium 2. Similarly, $H_{2}^{\left(-\right)}$ and $H_{3}^{\left(+\right)}$ are created through a reflection or transmission of the outgoing wave in medium 2. Solving these relations with respect to the unknown coefficients, we are led to Eq. (12) with the propagation constants *ψ*_2_ = *k*_2*ρ*_(*R*_2_ − *R*_1_) and *ψ* = *k*_1*ρ*_ *R*_1_ + *ψ*_2_ − *k*_3*ρ*_ *R*_2_. In a completely similar fashion, we can relate the coefficients *b*_*m*_(*θ*) inside and outside the capillary by propagating the transverse electric fields. In doing so, we simply have to replace the transverse magnetic reflection and transmission coefficients by the transverse electric ones.
